# Effects of a 4-Week Pilates Program on Cardiovascular, Metabolic, and Neuroendocrine Responses in Previously Sedentary Adult Women: An Age-Stratified Longitudinal Study

**DOI:** 10.3390/life16060945

**Published:** 2026-06-03

**Authors:** Ana Onu, Nela-Tatiana Balint, Gheorghe Balint, Alina-Mihaela Cristuta, Daniela Viorelia Matei

**Affiliations:** 1 Doctoral School, Grigore T. Popa University of Medicine and Pharmacy Iasi, 700115 Iasi, Romania; chirila.ana@d.umfiasi.ro (A.O.); daniela.matei@umfiasi.ro (D.V.M.); 2 Department of Physical Therapy, Micromedica Medlife Clinic, 610119 Piatra Neamt, Romania; 3 Faculty of Movement, Sports, and Health Sciences, “Vasile Alecsandri” University of Bacău, 600115 Bacău, Romania; cristuta.alina@ub.ro

**Keywords:** Pilates, exercise, cortisol, fasting glucose, cardiometabolic health, HPA axis, aging, rehabilitation, metabolic regulation, stress physiology

## Abstract

Background: Mind–body exercise interventions such as Pilates have been increasingly investigated for their potential to improve cardiovascular, metabolic, and neuroendocrine regulation; however, age-related physiological adaptations in previously sedentary women remain insufficiently characterized. Objective: To evaluate cardiovascular, metabolic, and neuroendocrine responses to a 4-week Pilates intervention in previously sedentary adult women using a single-arm longitudinal study design. Methods: This prospective longitudinal study included 30 women stratified into two age groups: 30–40 years (*n* = 15) and 50–60 years (*n* = 15). Participants completed a supervised 4-week Pilates program (3 sessions/week, moderate intensity). Resting heart rate (HR), systolic and diastolic blood pressure (SBP, DBP), body mass index (BMI), abdominal circumference (ABD), fasting blood glucose (GLU), and serum cortisol (CORT) were assessed at baseline (T0) and post-intervention (T1). Within-group changes were analyzed using paired Student’s *t*-tests, between-group differences using ANOVA, and associations between variables using Pearson correlation analysis. Results: Both groups demonstrated favorable cardiovascular, metabolic, and neuroendocrine adaptations following the intervention. In the 30–40 years group, significant reductions were observed in HR (Δ = −10.7 ± 3.2 bpm, *p* < 0.0001), BMI (Δ = −1.61 ± 0.4 kg/m^2^, *p* = 0.0050), and GLU (Δ = −9.7 ± 6.1 mg/dL, *p* = 0.0044). In the 50–60 years group, significant reductions were observed in SBP (Δ = −10.8 ± 3.1 mmHg, *p* = 0.0001), DBP (Δ = −7.1 ± 2.4 mmHg, *p* = 0.0001), GLU (Δ = −11.9 ± 7.3 mg/dL, *p* = 0.0051), and CORT (Δ = −54.9 ± 22 nmol/L, *p* = 0.0422). Between-group analyses demonstrated significantly greater reductions in SBP, DBP, BMI, and CORT, whereas changes in HR, ABD, and GLU did not differ significantly between age groups. Pearson correlation analysis revealed significant associations between cardiovascular, metabolic, and neuroendocrine adaptations, particularly between HR and BP responses in the younger group, and between BMI and GLU changes in the older group. Conclusions: A 4-week structured Pilates program induced favorable cardiovascular, metabolic, anthropometric, and neuroendocrine adaptations in previously sedentary adult women, with significant age-related differences observed for blood pressure, body mass index, and serum cortisol responses. These findings support Pilates as a multidimensional exercise intervention for cardiometabolic and stress regulation.

## 1. Introduction

The Pilates method represents a comprehensive system of muscle stretching and strengthening designed to promote a strong and balanced body, based on the philosophy of mind–body control [1]. Recent evidence has highlighted the potential of Pilates to improve physical fitness, balance, postural control, muscular endurance, psychological well-being, and quality of life across diverse populations [2,3,4,5]. In addition, Pilates has been associated with enhanced exercise adherence and participant engagement, supporting its value as a sustainable health-promoting intervention [6]. These benefits reflect the integrative mind–body nature of the Pilates method, which combines controlled movement, breathing, concentration, and neuromuscular coordination [1].

Pilates has gained increasing attention in physiotherapy, rehabilitation, and preventive health settings because of its broad spectrum of physical and psychological benefits [3,4,5,6,7,8]. Pilates exercises are specifically designed to activate postural and core musculature, improving stability, strength, flexibility, and overall body balance [9,10,11]. Furthermore, regular Pilates practice has been associated with reduced perceived stress, improved well-being, enhanced concentration, and better performance of daily activities [5,6,9]. Depending on the equipment used and the level of difficulty, Pilates exercises can be classified into several modalities, with mat Pilates relying primarily on body weight as resistance and often being preferred by older adults due to its low-impact nature [12].

Although various Pilates approaches have evolved, the method remains grounded in six fundamental principles: breathing, concentration, control, precision, centering, and flow [13]. These principles aim to enhance core strength, improve postural alignment, increase physical awareness, and promote efficient movement patterns [13,14]. As a result, Pilates has been proposed as a feasible and well-tolerated exercise modality for improving physical function and overall health, particularly among middle-aged and older adults [13,14].

Scientific literature identifies different types of physical activity that can be used to manage stress and its associated psychological symptoms [15]. Over time, physical training has proven to be an effective strategy for improving quality of life and promoting emotional well-being [16,17]. Recent meta-analysis results document moderate-to-large effects of supervised Pilates exercises on the physical well-being of clinical populations [18]. For example, Pilates has been validated as useful for reducing levels of depression, anxiety, and fatigue in patients with multiple sclerosis [19] and in patients with breast cancer [20]. Furthermore, this practice has led to a reduction in levels of depression, anxiety, and stress in overweight and obese individuals [21], as well as those suffering from chronic nonspecific back pain [22], tension-type headaches [23], and type 1 pediatric diabetes [24].

Cortisol is a steroid hormone synthesized from cholesterol that plays a key role during and after exercise, including stimulating gluconeogenesis and mobilizing and utilizing fatty acids for energy production [25]. Cortisol is widely used as a biomarker in assessing physiological responses to physical exertion in both elite athletes and clinical populations. Remarkably, increases in acute and basal cortisol levels in response to psychological stress are generally considered harmful to health, while increases associated with regular physical training are often considered adaptive and beneficial. It has been hypothesized that this apparent exercise-cortisol paradox involves mechanisms related to dopamine activity in the medial prefrontal cortex and glucocorticoid receptor regulation; however, most of the evidence supporting this hypothesis comes from animal studies (see Chen et al. [26] for a review). Although cortisol concentrations typically increase in response to intense exercise, this response is intensity-dependent and usually occurs only when a sufficient threshold of exercise is reached [27].

The main function of cortisol secretion in response to exercise is to increase the availability of substrates for metabolism, both during activity [28] and during recovery [29]. It has been shown that cortisol can then exhibit a “rebound” effect and remain depressed for 24–48 h after intense physical exertion [30]. This disruption of the hypothalamic–pituitary–adrenal (HPA) axis has implicated cortisol as a potential biomarker for diagnosing overtraining, although this effort has not yet yielded consistent results [31]. Furthermore, physical training can influence acute HPA responses to exercise by decreasing pituitary sensitivity to negative feedback [29] or increasing peripheral tissue sensitivity to cortisol [32]. There is also significant research on the role of cortisol in obesity [33].

Paradoxically, despite cortisol being a primary lipolytic hormone, increased body fat has been associated with elevated cortisol levels, suggesting that resting cortisol concentrations may be useful for monitoring the effectiveness of exercise and weight-loss interventions, particularly in obese individuals (McMurray and Hackney) [34].

Cortisol is one of the primary biomarkers of HPA-axis activity and plays a central role in the physiological adaptation to physical and psychological stress. As a glucocorticoid hormone synthesized by the adrenal cortex, cortisol regulates energy metabolism, glucose homeostasis, lipolysis, and immune function [35,36]. Acute exercise may transiently increase cortisol secretion depending on exercise intensity and duration, whereas regular physical activity has been associated with improved HPA-axis regulation and lower basal cortisol concentrations [37]. Recent evidence suggests that exercise interventions, including mind–body modalities, may reduce cortisol levels and improve physiological stress adaptation through neuroendocrine regulation [38]. In sedentary individuals, reductions in basal serum cortisol following structured exercise programs have been interpreted as markers of improved stress resilience and enhanced physiological efficiency [39]. Therefore, basal serum cortisol is a clinically relevant biomarker for assessing neuroendocrine adaptations induced by exercise interventions and may provide valuable insights into the relationships among physical activity, metabolic regulation, and stress physiology [36,37,38,39].

Based on the available evidence regarding the physiological effects of mind–body exercise interventions, we hypothesized that a 4-week structured Pilates program would induce favorable cardiovascular, metabolic, anthropometric, and neuroendocrine responses in previously sedentary adult women. We further hypothesized that the magnitude of these responses would differ between age groups, with older participants demonstrating distinct patterns of cardiovascular and neuroendocrine adaptation.

## 2. Materials and Methods

### 2.1. Study Design

This prospective, single-arm, longitudinal interventional study was conducted over five months, between October 2025 and February 2026, at Micromedica MedLife, Piatra Neamt Cliniq. Participant recruitment and intervention delivery were conducted in two consecutive waves between October 2025 and February 2026. Following baseline assessment, eligible participants were assigned to one of the two recruitment waves and subsequently completed the 4-week Pilates intervention under identical supervision and assessment procedures. The study was designed to investigate age-related cardiovascular, metabolic, and neuroendocrine adaptations to a structured Pilates exercise program in previously sedentary adult women.

A total of 30 female participants were enrolled and stratified into two age-based groups. The first group included 15 women aged 30–40 years, while the second group comprised 15 women aged 50–60 years. All participants completed the intervention and were included in the final statistical analysis, with no dropouts, adverse events, or protocol deviations recorded.

Participants were classified as sedentary if they had not participated in any regular structured exercise program during the previous three months. This threshold was selected to minimize the influence of residual training adaptations and to ensure a relatively homogeneous baseline level of physical inactivity before the intervention. Compliance with the pre-assessment instructions was verified through participant self-report immediately before each testing session. Participants were asked to confirm that they had abstained from caffeine, alcohol consumption, and vigorous physical activity according to the study requirements.

Given the exploratory physiological nature of the study and the objective of evaluating short-term adaptations to a structured Pilates intervention, a single-arm longitudinal design was employed. This design allowed the assessment of within-subject changes over time and provided preliminary information regarding cardiovascular, metabolic, anthropometric, and neuroendocrine responses to the intervention. Age stratification was incorporated to explore potential differences in adaptive responses between younger and older adult women. However, the absence of a non-exercising control group limits the ability to attribute observed changes exclusively to the intervention.

No formal a priori sample size calculation was performed. The study was designed as an exploratory pilot investigation intended to evaluate short-term cardiovascular, metabolic, anthropometric, and neuroendocrine responses to a structured Pilates intervention in previously sedentary women.

The study protocol was reviewed and approved by the Micromedica MedLife Scientific Research Ethics Committee (563 form 10 October 2025), and all participants provided written informed consent before enrollment, in accordance with the ethical principles of the Declaration of Helsinki.

### 2.2. Participants

Participant selection was conducted using predefined inclusion and exclusion criteria to ensure sample homogeneity and minimize confounding factors related to health status, habitual physical activity, and physiological variability.

The general inclusion criteria, applicable to both age groups, were designed to ensure participant safety and methodological consistency. Only female participants were enrolled, and all provided written informed consent before inclusion. Participants were required not to have participated in structured exercise programs during the previous three months. These criteria ensured that participants entered the intervention with a comparable low baseline level of structured physical activity, thereby reducing variability related to previous training adaptations.

General exclusion criteria were established to minimize confounding influences and ensure that outcomes could be attributed specifically to the Pilates intervention. Participants were excluded if they were male, engaged in other structured exercise programs, had neurological or cardiovascular disorders, or did not maintain consistent participation (i.e., <2 sessions per week).

Age-specific exclusion criteria were defined to ensure safety and avoid confounding effects from acute or unstable conditions. In the 30–40 years group, exclusion criteria included pregnancy or postpartum period <6 months, symptomatic intervertebral disk herniation without medical evaluation, and recent musculoskeletal trauma (<3 months). In the 50–60 years group, participants were excluded if they had severe osteoporosis with increased fracture risk, recent orthopedic surgery (<6 months), severe degenerative musculoskeletal conditions in the acute phase, or recent severe vertigo or marked postural instability.

Overall, these criteria ensured participant safety while allowing the observed outcomes to reflect true physiological adaptation to the Pilates intervention.

### 2.3. Pilates Intervention Protocol

All participants followed a standardized, supervised Pilates program designed to induce progressive physiological adaptation. The intervention lasted 4 weeks, with three sessions per week, each lasting 50–60 min. Exercise intensity was moderate and progressively adjusted according to individual tolerance and functional capacity.

During the 4-week intervention, participants were instructed to maintain their usual diet but to avoid alcohol, sugar-containing products, and sugar-sweetened beverages. No structured hypocaloric diet was prescribed, and caloric intake was not quantitatively monitored.

All Pilates sessions were conducted in the late afternoon (after 17:00) for both age groups. This standardized scheduling was implemented to minimize potential circadian influences on physiological and neuroendocrine responses, particularly cortisol variability, and to ensure consistency of exercise timing across the study population.

The program emphasized integrated neuromuscular control, including diaphragmatic breathing, core stabilization, spinal mobility, flexibility, postural alignment, balance training, and coordinated functional movement patterns. Exercise progression involved a gradual increase in motor control and movement complexity, with emphasis on movement quality, postural stability, and controlled muscle activation. All sessions were supervised to ensure proper technique and participant safety.

Exercise intensity was monitored using the Borg Rating of Perceived Exertion (RPE) scale. Participants were encouraged to maintain a moderate level of effort corresponding to values of 12–13 on the Borg 6–20 scale (“somewhat hard”), consistent with recommendations for moderate-intensity exercise.

Exercise progression was standardized across the intervention period. During Week 1, participants performed basic Pilates exercises focused on breathing control, core activation, and familiarization with movement patterns. During Week 2, exercise complexity was increased by incorporating longer movement sequences and greater trunk stabilization demands. In Week 3, additional balance and coordination challenges were introduced, together with increased repetitions. During Week 4, participants performed more complex integrated movement patterns requiring higher levels of postural control, coordination, and muscular endurance while maintaining proper technique and breathing control.

All participants followed a standardized mat-based Pilates program performed exclusively on the floor using light auxiliary equipment, including exercise balls, small stability balls, light dumbbells (0.5–1 kg), elastic resistance bands, wooden sticks, a Pilates ring (magic circle), and yoga blocks (cork or wooden rectangular supports). No Pilates apparatus (e.g., Reformer, Cadillac, or Chair) was used during the intervention, ensuring uniformity of exercise conditions and accessibility of the protocol.

### 2.4. Evaluation of Biological Parameters

Biological parameters were assessed to quantify metabolic regulation and neuroendocrine stress-axis activity in response to the structured Pilates intervention. Measurements were performed at baseline (T0) and after the 4-week intervention (T1) under strictly standardized pre-analytical conditions to ensure reproducibility and minimize biological variability.

Participants underwent venous blood sampling following an overnight fast of 12 h. To reduce acute confounding influences, they were instructed to refrain from vigorous physical activity, caffeine intake, alcohol consumption, and nicotine exposure for at least 24 h before testing. All blood samples were collected under strictly standardized conditions between 08:00 and 09:00 a.m. following an overnight fast, in order to control for the circadian rhythm of cortisol secretion and diurnal metabolic fluctuations. Venous blood sampling was performed by certified medical personnel under standardized clinical procedures to ensure sample integrity, participant safety, and pre-analytical reliability.

All biochemical analyses were performed in a certified clinical laboratory using standardized operating procedures and internal quality-control protocols. Duplicate measurements were not performed for individual samples; however, all analyses followed the manufacturer’s recommended calibration and quality-assurance procedures. Following clot formation, samples were centrifuged according to standardized laboratory protocols, and the serum/plasma was immediately processed for analysis at a certified clinical laboratory (Micromedica MedLife, Piatra Neamț, Romania), which operates under internal and external quality assurance programs.

Resting systolic and diastolic blood pressure were measured using an automated digital blood pressure monitor (Omron M6, Omron Healthcare Co., Ltd., Kyoto, Japan) according to standardized measurement procedures.

#### 2.4.1. Serum Cortisol (CORT)

CORT concentrations were quantified using an automated chemiluminescent microparticle immunoassay (CMIA) on the Abbott Architect platform (Abbott Laboratories, Abbott Park, IL, USA). This competitive immunoassay employs paramagnetic microparticles coated with anti-cortisol antibodies and acridinium-labeled cortisol conjugates for chemiluminescent detection. The assay is traceable to internationally recognized reference standards and calibrated according to manufacturer specifications.

The analytical sensitivity of the assay is approximately 5–10 ng/mL (≈14–28 nmol/L), within a broad analytical measurement range suitable for physiological and clinical cortisol concentrations. Analytical performance met clinical laboratory standards, with intra-assay and inter-assay coefficients of variation within validated precision limits (<5% and <8%, respectively). All samples from both time points were analyzed in the same laboratory using identical instrumentation and calibration procedures to minimize inter-assay variability. The use of automated chemiluminescent immunoassays for serum cortisol determination is widely validated in clinical endocrinology and research settings [40,41].

#### 2.4.2. Fasting Blood Glucose (GLU)

GLU concentrations were measured using the enzymatic hexokinase method on the Abbott Architect clinical chemistry analyzer (Abbott Laboratories, Abbott Park, IL, USA). This method is considered the reference standard for glucose quantification due to its high specificity, minimal interference, and excellent analytical precision. The assay operates through ATP-dependent phosphorylation of glucose to glucose-6-phosphate, followed by NADH generation, which is quantified photometrically.

The analytical sensitivity of the hexokinase method is approximately 1–2 mg/dL, with a coefficient of variation typically <2–3% under standardized laboratory conditions. Daily internal quality controls and calibration procedures were performed in accordance with laboratory accreditation standards to ensure analytical reliability and reproducibility. Glucose values were expressed in mg/dL. The hexokinase method is internationally recognized as the gold standard for plasma glucose measurement in both clinical diagnostics and metabolic research [42,43].

Together, these biomarkers were selected to provide a multidimensional evaluation of cardiometabolic regulation and HPA axis adaptation to moderate-intensity exercise. The combination of automated CMIA and reference enzymatic methodology ensures high analytical accuracy, traceability, and comparability with international clinical research standards.

### 2.5. Anthropometric and Cardiovascular Assessment

Anthropometric and cardiovascular parameters were evaluated under standardized resting conditions at both time points. BMI was calculated as body weight divided by height squared (kg/m^2^). Abdominal circumference (ABD) was measured at the midpoint between the lower costal margin and the iliac crest using a non-elastic measuring tape.

Resting heart rate (HR) and arterial BP were recorded after a 10 min seated rest using validated automated monitoring devices. All measurements were performed by trained personnel following standardized procedures to ensure reproducibility and minimize observer bias.

These parameters were included to complement biological assessment and provide an integrated evaluation of cardiovascular regulation and metabolic status.

### 2.6. Statistical Analysis

Statistical analyses were performed using IBM SPSS Statistics, Version 29 (IBM Corp., Armonk, NY, USA). The normality of data distribution was assessed using the Shapiro–Wilk test. All variables demonstrated approximately normal distributions (*p* > 0.05), supporting the use of parametric statistical methods.

Within-group changes were analyzed using paired Student’s *t*-tests. To evaluate age-related differences in response to the intervention, mixed-design ANOVA was performed with Time (T0 vs. T1) as the within-subject factor and Age Group (30–40 vs. 50–60 years) as the between-subject factor. Pearson correlation analysis was used to assess associations between physiological adaptations. To evaluate age-related differences in response to the intervention, a mixed-design ANOVA was performed with Time (baseline vs. post-intervention) as the within-subject factor and Age Group (30–40 years vs. 50–60 years) as the between-subject factor. Main effects and Time × Group interaction effects were examined for all outcome variables. Pearson correlation analysis was subsequently used to assess associations between physiological responses.

Before statistical analysis, the assumptions underlying parametric testing were evaluated. Normality of data distribution was assessed using the Shapiro–Wilk test, while homogeneity of variance between groups was examined using Levene’s test. All variables met the assumptions required for parametric analyses (*p* > 0.05).

## 3. Results

### 3.1. Baseline Characteristics of the Study Population

All 30 participants completed the intervention protocol and were included in the final analysis. The cohort was divided into two age-stratified groups (30–40 years and 50–60 years), with no dropouts or adverse events recorded during the study period, confirming both the feasibility and tolerability of the intervention.

At baseline (T0), clear differences in physiological and metabolic status were observed between the two age groups. The 50–60-year-old group exhibited consistently higher values for HR (92.333 ± 7.098 bpm vs. 87.733 ± 5.496 bpm), SBP (139.333 ± 4.746 mmHg vs. 129.800 ± 4.092 mmHg), DBP (88.067 ± 2.685 mmHg vs. 82.467 ± 2.326 mmHg), BMI (29.880 ± 1.267 kg/m^2^ vs. 29.320 ± 1.507 kg/m^2^), ABD (91.200 ± 6.372 cm vs. 88.467 ± 6.278 cm), GLU (113.800 ± 13.380 mg/dL vs. 108.200 ± 10.910 mg/dL), and CORT (415.067 ± 63.042 nmol/L vs. 351.400 ± 68.128 nmol/L) compared to the younger group. This pattern may reflect baseline age-related differences in cardiovascular, metabolic, and neuroendocrine regulation, even in the absence of overt pathology (Table 1 and Table 2).

In contrast, the younger group presented lower baseline values across all investigated parameters, indicating a comparatively more favorable physiological profile at study entry. Despite these differences, variability within each group remained acceptable, supporting the internal consistency and homogeneity of the study sample.

Baseline and post-intervention values for the 30–40 years group are presented in Table 1, while the corresponding data for the 50–60 years group are shown in Table 2. The intervention effects are described in detail in Section 3.2.

### 3.2. Effects of the Pilates Intervention

After the 4-week intervention, both groups demonstrated consistent improvements across the investigated cardiovascular, anthropometric, metabolic, and neuroendocrine parameters (Table 3).

In the younger group (30–40 years), the most evident changes were observed in HR, which showed a significant reduction following the intervention (Δ = −10.7 ± 3.2 bpm, *p* < 0.0001). SBP and DBP also decreased significantly, although to a lesser extent compared to the older group. BMI demonstrated a moderate but statistically significant reduction (Δ = −1.63 ± 0.4 kg/m^2^, *p* = 0.0050). GLU levels also decreased significantly (Δ = −9.7 ± 6.1 mg/dL, *p* = 0.0044). Although CORT and ABD showed reductions after the intervention, these changes did not reach statistical significance.

In the older group (50–60 years), improvements were more pronounced at the vascular and neuroendocrine levels. SBP and DBP demonstrated larger reductions compared to the younger participants (ΔSBP = −10.8 ± 3.1 mmHg; ΔDBP = −7.1 ± 2.4 mmHg; both *p* = 0.0001). In parallel, CORT concentrations showed a significant decrease following the intervention (Δ = −54.9 ± 22 nmol/L, *p* = 0.0422). Similarly, GLU levels decreased significantly (Δ = −11.9 ± 7.3 mg/dL, *p* = 0.0051).

Significant Time × Group interactions and serum cortisol indicated age-dependent adaptation patterns.

### 3.3. Pearson Correlation Analysis

The Pearson correlation analysis revealed distinct interaction patterns between cardiovascular, anthropometric, metabolic, and neuroendocrine adaptations in the two age groups (Table 4 and Table 5).

In the younger group (30–40 years), strong positive correlations were observed between changes in HR and SBP (*r* = 0.82, *p* = 0.0002), as well as DBP (*r* = 0.93, *p* < 0.0001). Additionally, systolic and diastolic BP changes were highly correlated (*r* = 0.95, *p* < 0.0001). CORT variation demonstrated significant negative correlations with SBP (*r* = −0.56, *p* = 0.028) and DBP (*r* = −0.58, *p* = 0.022), while a positive association was identified between CORT and BMI changes (*r* = 0.56, *p* = 0.029).

In the older group (50–60 years), significant positive correlations were identified between systolic and diastolic BP responses (*r* = 0.61, *p* = 0.015), DBP and BMI changes (*r* = 0.63, *p* = 0.011), and BMI and GLU variation (*r* = 0.53, *p* = 0.043). These findings suggest a closer interaction between anthropometric and metabolic adaptations in older participants following the intervention.

## 4. Discussion

The present study investigated age-related physiological adaptations to a structured 4-week Pilates program in previously sedentary adult women, with a particular focus on cardiovascular regulation, metabolic profile, and neuroendocrine stress-axis activity. The results demonstrated consistent multisystem improvements, with distinct age-dependent adaptation patterns.

Both age groups showed significant improvements following the intervention; however, the nature of these adaptations differed. Between-group comparisons indicated significantly greater reductions in SBP, DBP, and serum CORT concentrations among older participants. In contrast, changes in HR, ABD, and GLU were comparable between age groups, suggesting that not all physiological adaptations were age dependent. These differences likely reflect baseline physiological disparities, with older individuals presenting a higher cardiometabolic burden and, consequently, a greater potential for adaptive change.

The reduction in HR observed in both groups is consistent with the well-documented effects of regular moderate-intensity exercise on autonomic balance. Exercise training has been associated with improvements in autonomic function, although autonomic regulation was not directly assessed in the present study. The slightly greater HR response observed in younger participants may suggest differences in cardiovascular responsiveness between age groups; however, autonomic function was not directly evaluated. Therefore, the observed reductions in blood pressure should be interpreted as responses occurring during the intervention period rather than definitive causal effects of Pilates training.

In parallel, significant reductions in SBP were observed, particularly in the older group (*p* < 0.0001), supporting the role of exercise in vascular regulation. Regular physical activity is known to improve endothelial function, reduce arterial stiffness, and enhance nitric oxide bioavailability, thereby contributing to BP reduction [3,4]. The larger decrease observed in the older group may be attributed to higher baseline BP values and increased vascular stiffness, which allow for a greater magnitude of improvement following intervention.

The reduction in BMI observed in the younger group should be interpreted with caution. Although Pilates may contribute to improved body composition through increased energy expenditure, neuromuscular activation, and improved adherence to healthy behaviors, the magnitude of BMI reduction observed over four weeks is unlikely to be explained by Pilates training alone. Importantly, during the intervention period, participants were instructed to avoid alcohol, sugar-containing products, and sugar-sweetened beverages while otherwise maintaining their usual diet. Therefore, the observed anthropometric changes may reflect the combined influence of structured Pilates training and partial dietary modification, particularly reduced intake of added sugars and caloric beverages [5].

The observed decrease in GLU further supports improved metabolic regulation following the intervention. Exercise enhances insulin sensitivity, promotes glucose uptake in skeletal muscle, and improves overall glycemic control [6,7]. In the present study, GLU levels decreased significantly in both groups (*p* < 0.005 in the older group; *p* < 0.004 in the younger group), suggesting that individuals with higher baseline metabolic dysfunction may derive greater metabolic benefits from structured exercise interventions.

A central finding in the present study is the significant reduction in serum CORT concentrations, particularly in the older group (*p* < 0.05). Cortisol is a key biomarker of HPA axis activity and plays an essential role in energy metabolism, glucose regulation, and physiological adaptation to stress. While acute bouts of exercise may transiently increase CORT secretion, repeated exposure to moderate-intensity exercise has been associated with improved neuroendocrine efficiency and reduced basal CORT concentrations. The reduction observed in the present study suggests lower basal CORT concentrations following the intervention; however, direct conclusions regarding HPA-axis regulation cannot be drawn from a single morning CORT measurement. The larger decrease observed in older participants may reflect a higher baseline neuroendocrine burden and a greater adaptive reserve following the intervention [8,9].

This finding is consistent with previous studies reporting reductions in basal cortisol concentrations following regular physical activity [10,11]. The larger decrease observed in older participants may be partially related to their higher baseline cortisol concentrations; however, this interpretation remains speculative and should be confirmed in future studies using formal adjustment analyses [12].

An important aspect that should be considered when interpreting the present findings is the relatively short duration of the intervention. The Pilates program consisted of only 12 supervised sessions performed over 4 weeks. Although significant improvements were observed in several cardiovascular, metabolic, and neuroendocrine parameters, these changes should be interpreted as short-term physiological responses rather than evidence of permanent adaptation. Previous studies investigating Pilates and other exercise modalities have commonly employed intervention periods of 8–12 weeks or longer, allowing more extensive cardiovascular, metabolic, and hormonal remodeling. Therefore, while the present results suggest that measurable benefits may occur even after a relatively brief intervention, longer-term studies are necessary to determine the durability, magnitude, and clinical significance of these adaptations.

The Pearson correlation analysis revealed significant associations between cardiovascular, anthropometric, metabolic, and neuroendocrine adaptations following the intervention. In the younger group, changes in HR were strongly correlated with both SBP (*r* = 0.82, *p* < 0.001) and DBP (*r* = 0.93, *p* < 0.001), suggesting coordinated cardiovascular responses. In addition, CORT changes demonstrated significant negative correlations with SBP (*r* = −0.56, *p* < 0.05) and DBP (*r* = −0.58, *p* < 0.05), while showing a positive association with BMI variation (*r* = 0.56, *p* < 0.05). These findings suggest that neuroendocrine adaptation may be closely linked to cardiovascular and anthropometric regulation following exercise.

In the older group, the positive association between BMI and GLU changes (*r* = 0.53, *p* < 0.05) highlights the relationship between adiposity and metabolic regulation. Furthermore, DBP changes were positively correlated with BMI variation (*r* = 0.63, *p* = 0.011), suggesting that improvements in vascular regulation may be associated with reductions in body mass. A significant correlation was also observed between SBP and DBP responses (*r* = 0.61, *p* = 0.015), indicating coordinated hemodynamic adaptation following the intervention. Although ABD and CORT changes did not demonstrate strong independent correlations with GLU variation, the overall pattern suggests an interaction between anthropometric status, neuroendocrine regulation, and metabolic control.

Together, these findings support the hypothesis that Pilates-induced physiological adaptations involve interconnected cardiovascular, anthropometric, metabolic, and neuroendocrine mechanisms, with distinct age-dependent response profiles.

Pilates, as a mind–body exercise modality, integrates controlled breathing, concentration, and neuromuscular coordination, all of which are known to influence autonomic and stress-related responses. Previous studies have shown that Pilates can reduce psychological stress, anxiety, and depressive symptoms while improving overall well-being [13,14,15,16]. The reduction in CORT observed in the present study supports the hypothesis that such interventions may attenuate physiological stress-axis activation and enhance stress resilience.

The differential response observed between age groups suggests that exercise-induced adaptations are influenced by baseline physiological status. While younger individuals primarily exhibited improvements in autonomic regulation, older individuals experienced more substantial metabolic and neuroendocrine benefits. These age-related differences may provide a basis for future research exploring individualized exercise approaches, although the present data are insufficient to support specific prescription recommendations.

Although several correlations reached statistical significance, most were of moderate magnitude and should be interpreted cautiously. Nevertheless, these associations may suggest potentially meaningful interactions between cardiovascular, metabolic, and neuroendocrine responses to Pilates training, providing a basis for future investigations in larger populations.

From a clinical perspective, the present findings suggest that structured Pilates programs may be associated with favorable cardiovascular, metabolic, and neuroendocrine responses; however, these observations require confirmation in larger controlled studies. The integrative nature of the observed adaptations underscores the importance of considering exercise as a systemic intervention that targets interconnected physiological pathways rather than isolated outcomes.

Several limitations should be considered when interpreting the present findings. First, the single-arm design without a parallel non-exercising control group limits the internal validity of the study and precludes definitive causal inference. Consequently, the observed physiological changes cannot be attributed exclusively to the Pilates intervention, as potential influences from the Hawthorne effect, spontaneous lifestyle modifications, seasonal factors, or regression to the mean cannot be excluded.

Second, the relatively small sample size reduces statistical power and limits the generalizability of the findings. Third, CORT was assessed using a single fasting morning serum measurement, which reflects basal HPA-axis activity but does not capture the complete diurnal rhythm of cortisol secretion. Furthermore, dietary intake was not quantitatively monitored, although participants were instructed to avoid alcohol, sugar-containing products, and sugar-sweetened beverages during the intervention. Therefore, the contribution of dietary changes to the observed anthropometric and metabolic adaptations cannot be fully separated from the effects of exercise. Finally, the inclusion of only female participants limits the extrapolation of these findings to male populations. Additionally, the intervention lasted only four weeks and included twelve supervised sessions. Although significant physiological changes were detected, the short duration limits conclusions regarding the long-term stability and persistence of the observed adaptations.

Accordingly, the present results should be considered preliminary and hypothesis-generating. Larger randomized controlled trials with longer follow-up periods are required to confirm these findings and establish causal relationships between Pilates training and physiological adaptation. Furthermore, physical activity performed outside the supervised sessions was not objectively monitored, and body composition was assessed using BMI and abdominal circumference rather than direct methods such as bioelectrical impedance analysis or dual-energy X-ray absorptiometry.

Future studies should include larger randomized controlled designs, incorporate longitudinal follow-up, and explore sex-specific responses to mind–body exercise interventions. Moreover, integrating additional biomarkers of inflammation and autonomic function could further elucidate the mechanisms underlying exercise-induced physiological adaptation.

Importantly, the observed correlations should not be interpreted as evidence of causal relationships. Rather, they identify potential associations between physiological responses that may warrant further investigation in larger longitudinal and mechanistic studies.

## 5. Conclusions

The present study suggests that a structured 4-week Pilates intervention may promote favorable cardiovascular, metabolic, anthropometric, and neuroendocrine responses in previously sedentary adult women.

Younger participants primarily exhibited greater reductions in resting heart rate and improvements in anthropometric parameters. In contrast, older participants demonstrated stronger vascular, metabolic, and neuroendocrine responses, including larger reductions in blood pressure, fasting glucose, and serum cortisol concentrations.

The observed associations between cardiovascular, metabolic, and neuroendocrine variables suggest that Pilates may be associated with concurrent changes across multiple physiological domains. These findings indicate that even a relatively short-duration Pilates program may contribute to favorable cardiometabolic and stress-related responses.

From a clinical perspective, Pilates may represent a feasible non-pharmacological intervention for promoting physiological health in sedentary middle-aged women. However, given the exploratory single-arm design and the absence of a non-exercising control group, the findings should be considered preliminary and interpreted with caution.

Future randomized controlled studies with larger sample sizes and longer follow-up periods are needed to confirm these findings and further clarify the mechanisms underlying exercise-induced physiological responses.

## Figures and Tables

**Table 1:** Physiological and metabolic changes following the 4-week Pilates intervention in the 30–40 years group.

Parameters	Initial	Final	*p*
HR (bpm)	87.733 ± 5.496	77.067 ± 4.758	<0.0001
SBP (mmHg)	129.800 ± 4.092	123.267 ± 2.314	<0.0001
DBP (mmHg)	82.467 ± 2.326	77.733 ± 1.534	<0.0001
BMI	29.320 ± 1.507	27.713 ± 1.382	0.0050
ABD (cm)	88.467 ± 6.278	84.733 ± 6.017	0.1075
CORT (nmol/L)	351.400 ± 68.128	318.867 ± 59.737	0.1753
GLU (mg/dL)	108.200 ± 10.910	98.533 ± 5.194	0.0044

Data are expressed as mean ± SD. HR—heart rate; SBP—systolic blood pressure; DBP—diastolic blood pressure; BMI—body mass index; ABD—abdominal circumference.

**Table 2:** Physiological and metabolic changes following the 4-week Pilates intervention in the 50–60 years group.

Parameters	Initial	Final	*p*
HR (bpm)	92.333 ± 7.098	83.267 ± 6.692	0.0012
SBP (mmHg)	139.333 ± 4.746	128.533 ± 4.068	0.0001
DBP (mmHg)	88.067 ± 2.685	80.933 ± 1.944	0.0001
BMI	29.880 ± 1.267	29.213 ± 1.141	0.1411
ABD (cm)	91.200 ± 6.372	87.333 ± 6.172	0.1025
CORT (nmol/L)	415.067 ± 63.042	360.133 ± 77.541	0.0422
GLU (mg/dL)	113.800 ± 13.380	101.933 ± 7.035	0.0051

Data are expressed as mean ± SD. HR—heart rate; SBP—systolic blood pressure; DBP—diastolic blood pressure; BMI—body mass index; ABD—abdominal circumference.

**Table 3:** Changes after 4-week intervention (Δ = T1 − T0).

Parameter	30–40 Years	50–60 Years	Time × Group *p*
Δ HR (bpm)	10.7 ± 3.2	9.1 ± 3.5	0.246
Δ SBP (mmHg)	6.5 ± 2.8	10.8 ± 3.1	<0.001
Δ DBP (mmHg)	4.7 ± 2.1	7.1 ± 2.4	<0.001
Δ BMI (kg/m^2^)	1.61 ± 0.4	0.67 ± 0.5	<0.001
Δ ABD (cm)	3.7 ± 1.8	3.9 ± 2.0	0.788
Δ CORT (nmol/L)	32.5 ± 18	54.9 ± 22	0.015
Δ GLU (mg/dL)	9.7 ± 6.1	11.9 ± 7.3	0.487

**Table 4:** Pearson correlation matrix for physiological and metabolic changes in the 30–40 years group.

Variables	*HR	*SBP	*DBP	*BMI	*ABD	*Cortisol	*Glucose
*HR	—	0.8249 **	0.9311 **	0.0817	0.2144	−0.4844	0.1761
*SBP	0.0002	—	0.9510 **	−0.2566	0.1834	−0.5648 *	0.0513
*DBP	<0.0001	<0.0001	—	−0.1019	0.2165	−0.5855 *	0.0952
*BMI	0.7723	0.3559	0.7178	—	0.2696	0.5633 *	0.1267
*ABD	0.4429	0.5130	0.4382	0.3312	—	0.1339	0.2482
*CORT	0.0673	0.0283	0.0218	0.0288	0.6342	—	0.1951
*GLU	0.5301	0.8561	0.7359	0.6527	0.3723	0.4860	—

Values in the upper matrix represent Pearson correlation coefficients (*r*), while values in the lower matrix represent *p* values (two-tailed). * *p* < 0.05; ** *p* < 0.001. *HR—difference between final and initial value of HR. *SBP—difference between final and initial value of SBP.

**Table 5:** Pearson correlation matrix for physiological and metabolic changes in the 50–60 years group.

Variables	*HR	*SBP	*DBP	*BMI	*ABD	*Cortisol	*Glucose
*HR	—	−0.0499	0.0532	−0.0310	−0.2095	0.3670	0.0618
*SBP	0.8598	—	0.6135 *	0.1487	0.1143	0.3008	−0.1140
*DBP	0.8506	0.0150	—	0.6346 *	0.0121 *	0.3826	−0.4438
*BMI	0.9127	0.5969	0.0110	—	−0.2849	0.4285	0.5287 *
*ABD	0.4535	0.6850	0.9660	0.3034	—	−0.3441	0.2817
*CORT	0.1784	0.2760	0.1593	0.1110	0.2092	—	−0.1564
*GLU	0.8268	0.6857	0.0975	0.0427	0.3091	0.5779	—

Values in the upper matrix represent Pearson correlation coefficients (*r*), while values in the lower matrix represent *p* values (two-tailed). * *p* < 0.05; *HR—difference between final and initial value of HR. *SBP—difference between final and initial value of SBP.

## Data Availability

The original contributions presented in this study are included in the article. Further inquiries can be directed to the corresponding author.

## Abbreviations

The following abbreviations are used in this manuscript:

ABD	Abdominal circumference
BMI	Body mass index
CORT	Cortisol
DBP	Diastolic blood pressure
GLU	Fasting blood glucose
HPA	Hypothalamic–pituitary–adrenal
HR	Resting heart rate
SBP	Systolic blood pressure
